# The effectiveness of couples’ relationship enrichment counseling on sexual function, satisfaction, shame and self-compassion in Iranian women with type 2 diabetes

**DOI:** 10.1192/j.eurpsy.2026.11453

**Published:** 2026-07-31

**Authors:** M. Sharifi, C. A. Lewis, M. Firoozi, S. Khani, P. Afkari

**Affiliations:** 1 Psychology, Leeds Trinity University, Leeds, United Kingdom; 2 Psychology, Islamic Azad University, Bandargaz Branch, Bandargaz; 3 Psychology; 4 Psychology and Educational Sciences, University of Tehran; 5 Psychology, Payam-e-noor University, Tehran, Iran, Islamic Republic Of

## Abstract

**Introduction:**

There is presently limited information on the sexual experiences of women with type 2 diabetes (T2DM) in Iran.

**Objectives:**

This study aimed to determine the effectiveness of couples’ relationship enrichment counseling on sexual function and sexual satisfaction, sexual shame and sexual self-compassion in women with T2DM.

**Methods:**

A quasi-experimental design with pretest-posttest and control group was employed. A purposive sample of 68 Iranian women with T2DM was randomly assigned to two groups: an intervention group (n = 34) and a control group (n = 34). The intervention group received six 90-minute sessions of couples’ relationship enrichment counseling, while the control group did not receive any training. Data were collected using self-report scales on women’s Sexual Function Index, Sexual Satisfaction Questionnaire, Sexual Shame, and Sexual Self-Compassion. Then they were analyzed using multivariate analysis of variance with repeated measurements.

**Results:**

Before the intervention, there was no significant difference between the intervention and control groups in the scores obtained by the scales (p >.05). After the intervention, the mean scores on all scales except sexual shame were significantly different between the two groups, with the intervention group scoring significantly higher (p < .05).

**Conclusions:**

Therefore, the findings provide evidence that couples’ relationship enrichment counseling may be useful for improving sexual function, sexual satisfaction, and sexual self-compassion among women with T2DM in Iran.

**Disclosure of Interest:**

None Declared

